# The Intersection of Chronic Disease and Economic Hardship in Rural African American Communities

**DOI:** 10.1093/geroni/igaf122.3222

**Published:** 2025-12-31

**Authors:** Idethia Shevon Harvey

**Affiliations:** University of Missouri, Columbia, Missouri, United States

## Abstract

Introduction Multiple chronic conditions disproportionately affect rural African Americans who face higher prevalence and worse outcomes for arthritis, diabetes, and hypertension. Those living in challenging environments experience higher rates of chronic comorbidities due to the interaction between rural limitations and persistent structural factors. The research investigates using syndemic theory to forge new ground in rural minority health research. Methods Semi-structured interviews and photo-documentation were conducted among African Americans (mean age = 63.2, SD + 9.93 years) with long-term diabetes (mean diagnosis = 14.7, SD + 10.8 years) and hypertension (91.3%). Quantitative analysis included univariate and bivariate statistics describing demographics and self-management behaviors by composite score (0 – 6) counting depression, hypertension, arthritis, smoking, obesity, and poverty. Qualitative methods included semi-structured interviews, photo-documentation, thematic content analysis, and critical visual methodology. Triangulation occurred when photographic themes aligned with interview themes. Results Participants with higher syndemic scores (4.31, SD + 0.48) showed lower days of health eating, physical activity, and glucose testing adherence. Qualitative analysis revealed two themes: “chronicity of poverty” and “contextual factors affecting health management.” Limited economic resources not only discouraged positive diabetes management but triggered clustering of health complications, supporting syndemic theory that individuals with diabetes experience magnified health-related and socioenvironmental burdens. Discussion This syndemic approach provides a novel perspective for understanding contributing factors influencing health in rural African American communities. A comprehensive approach addressing biological and social elements is necessary to reduce disease burden, improve patient-centered care, and develop targeted programs supporting self-management practices in these communities.

